# Intensive-Dose Tinzaparin in Hospitalized COVID-19 Patients: The INTERACT Study

**DOI:** 10.3390/v14040767

**Published:** 2022-04-07

**Authors:** Karolina Akinosoglou, Christos Savopoulos, Abraham Pouliakis, Charalampos Triantafyllidis, Eleftherios Markatis, Foteini Golemi, Angelos Liontos, Charikleia Vadala, Ilias C. Papanikolaou, Vasiliki Dimakopoulou, Panagiotis Xarras, Katerina Varela, Georgia Kaiafa, Athanasios Mitsianis, Anastasia Chatzistamati, Efthalia Randou, Spyridon Savvanis, Maria Pavlaki, Georgios Efraimidis, Vasileios Samaras, Dimitrios Papazoglou, Alexandra Konstantinidou, Periklis Panagopoulos, Haralampos Milionis, on behalf of the INTERACT Study Group

**Affiliations:** 1 Internal Medicine Department, University General Hospital of Patras, 265 04 Rio, Greece; dimakopoulou.vasilina@gmail.com; 2 1st Propaedeutic Department of Internal Medicine, University General Hospital of Thessaloniki “AXEPA”, 546 21 Thessaloniki, Greece; chrisavopoulos@gmail.com (C.S.); gdkaiafa@yahoo.gr (G.K.); 3 2nd Department of Pathology, National and Kapodistrian University of Athens, “ATTIKON” University Hospital, 124 62 Chaidari, Greece; apou1967@gmail.com; 4 1st Pulmonology Department, General Hospital of Kavala “Saint Sylas”, 655 00 Kavala, Greece; harrydoc2000@yahoo.gr (C.T.); konstantinidoua4@gmail.com (A.K.); 5 Pulmonary Department, General Hospital of Corfu “Saint Eirini”, 491 00 Kontokali, Greece; lefte_mark83@yahoo.gr (E.M.); icpapanikolaou@hotmail.com (I.C.P.); 6 Pathology Department, Argolida General Hospital–Argos Hospital Unit, 212 00 Argos, Greece; foteinh_golemi@hotmail.com (F.G.); mar_paul300@yahoo.gr (M.P.); 7 Department of Internal Medicine, Faculty of Health Sciences, School of Medicine, University of Ioannina, 451 10 Ioannina, Greece; angelosliontos@gmail.com (A.L.); hmilioni@uoi.gr (H.M.); 8 4th Internal Medicine Department, Athens General Hospital “Evangelismos”, 106 76 Athens, Greece; hara.vadala@gmail.com; 9 Internal Medicine Department, General Hospital of Kozani “Mamatseio”, 501 00 Kozani, Greece; panosxarras@gmail.com (P.X.); vag_roundos@yahoo.gr (E.R.); 10 Pulmonary Department, General Hospital of Patras “Saint Andreas”, 265 04 Patra, Greece; alerav99@yahoo.gr; 11 Internal Medicine Department, General Hospital of Ptolemaida “Bodosakeio”, 50 200 Ptolemaida, Greece; athemit@gmail.com; 12 Internal Medicine Department, General Hospital of Kastoria, 521 00 Kastoria, Greece; 5755anastasiachtz@gmail.com (A.C.); billsamaras@gmail.com (V.S.); 13 Internal Medicine Department, General Hospital of Athens “Elpis”, 106 76 Athens, Greece; savvanis@live.com; 14 Internal Medicine Department, General Hospital of Patras “Saint Andreas”, 263 32 Patra, Greece; efrgeorge@gmail.com; 15 Infectious Diseases Unit, 2nd Department of Internal Medicine, University General Hospital of Alexandroupolis, 681 00 Alexandroupoli, Greece; dpapazog@med.duth.gr (D.P.); ppanago@med.duth.gr (P.P.)

**Keywords:** COVID-19, SARS-CoV-2, coronavirus, thrombosis, thromboprophylaxis, low molecular weight heparins, tinzaparin

## Abstract

(1) Background: It is well-established that coronavirus disease-2019 (COVID-19) is highly pro-inflammatory, leading to activation of the coagulation cascade. COVID-19-induced hypercoagulability is associated with adverse outcomes and mortality. Current guidelines recommend that hospitalized COVID-19 patients should receive pharmacological prophylaxis against venous thromboembolism (VTE). (2) INTERACT is a retrospective, phase IV, observational cohort study aiming to evaluate the overall clinical effectiveness and safety of a higher than conventionally used prophylactic dose of anticoagulation with tinzaparin administered for VTE prevention in non-critically ill COVID-19 patients with moderate disease severity. (3) Results: A total of 705 patients from 13 hospitals in Greece participated in the study (55% men, median age 62 years). Anticoagulation with tinzaparin was initiated immediately after admission. A full therapeutic dose was received by 36.3% of the participants (mean ± SD 166 ± 33 IU/Kgr/day) and the remaining patients (63.9%) received an intermediate dose (mean ± SD 114 ± 22 IU/Kgr/day). The median treatment duration was 13 days (Q1–Q3: 8–20 days). During the study (April 2020 to November 2021), 14 thrombotic events (2.0%) were diagnosed (i.e., three cases of pulmonary embolism (PE) and 11 cases of deep venous thrombosis, DVT). Four bleeding events were recorded (0.6%). In-hospital death occurred in 12 patients (1.7%). Thrombosis was associated with increasing age (median: 74.5 years, Q1–Q3: 62–79, for patients with thrombosis vs. 61.9 years, Q1–Q3: 49–72, *p* = 0.0149), increased D-dimer levels for all three evaluation time points (at admission: 2490, Q1–Q3: 1580–6480 vs. 700, Q1–Q3: 400–1475, *p* < 0.0001), one week ± two days after admission (3510, Q1–Q3: 1458–9500 vs. 619, Q1–Q3: 352–1054.5, *p* < 0.0001), as well as upon discharge (1618.5, Q1–Q3: 1010–2255 vs. 500, Q1–Q3: 294–918, *p* < 0.0001). Clinical and laboratory improvement was affirmed by decreasing D-dimer and CRP levels, increasing platelet numbers and oxygen saturation measurements, and a drop in the World Health Organization (WHO) progression scale. (4) Conclusions: The findings of our study are in favor of prophylactic anticoagulation with an intermediate to full therapeutic dose of tinzaparin among non-critically ill patients hospitalized with COVID-19.

## 1. Introduction

A prothrombotic state attributable to a cytokine storm induced by severe acute respiratory syndrome coronavirus 2 (SARS-CoV-2) and resulting in activation of the coagulation cascade is an established feature of coronavirus disease 2019 (COVID-19). A strong inflammatory response which is orchestrated by various inflammatory mediators ends in activation of mononuclear cells. These cells express tissue factor (TF) on their surface, leading to thrombin generation and subsequent fibrinogen-to-fibrin conversion and clot formation. At the same time, SARS-CoV-2 can directly infect and damage endothelial cells, causing massive release of plasminogen activators and von Willebrand factor (vWF) [1]. Clinically, thrombin generation, fibrin formation, and fibrinolysis manifest as venous thromboembolism (VTE), arterial thrombosis events (ATE), and disseminated intravenous coagulation (DIC) [1].

Coagulopathy is reflective of more severe disease and adverse prognosis [2]. A significant number of patients with COVID-19 require single or multiple organ support on the intensive care unit (ICU); this is estimated to comprise between 12% and 17% of patients [3,4,5,6]. However, most patients who are hospitalized with COVID-19 are moderately ill and do not initially require organ support in an intensive care unit [7,8]. Limited therapies are available to prevent progression to organ failure and death among moderately ill patients. COVID-19 severity can be classified based on parameters such as age, previous VTE, BMI > 30 kg/m^2^, ICU hospitalization, and hypercoagulability (fibrinogen > 800 mg/dL, D-dimers > 3000 ng/mL). Four different levels of thromboembolic risk can be determined, such as low, intermediate, high, and very high risk, necessitating the use of thromboprophylaxis in COVID-19 patients, considering the renal, hepatic function, and the co-administered drugs against COVID-19 infection.

Current guidelines recommend that hospitalized patients with COVID-19 should receive pharmacological prophylaxis against VTE, in the absence of contraindications [9,10,11]. Systemic anticoagulation is associated with improved in-hospital survival in intubated patients or patients with severe COVID-19 in multiple cohorts [12,13].

Low molecular weight heparins (LMWH), in addition to their well-known anticoagulant properties, appear to have additional antiviral and anti-inflammatory effects that may potentially be beneficial for hospitalized COVID-19 patients [14,15].

Though international and national guidelines state that all hospitalized patients with COVID-19 should receive pharmacologic thromboprophylaxis, the rising incidence of thrombotic complications in COVID-19 patients has led a lot of hospitals to adopt the strategy of increasing the dose of anticoagulation for prophylaxis to “intermediate” or “full therapeutic” doses using a risk-adapted strategy with increased doses administration based on factors associated with increased risk. Clinicians weigh the benefits and risks of therapeutic anticoagulation in terms of thrombosis and major bleeding risk for individual patients. It has also been hypothesized that anticoagulation with heparin administered at doses higher than conventionally used for venous thromboprophylaxis may improve the course of COVID-19 disease and patients’ outcomes. We should keep in mind that Heparin Resistance (HR) is common among COVID-19 patients in the ICU and increases in parallel to the overall illness severity. There are three causes of HR: pseudo-heparin resistance (high levels of factor VIII and/or fibrinogen artificially lower the PTT level); ATIII deficiency, which does not seem a significant driver of HR; and low heparin concentration due to acute-phase proteins, which is probably the primary cause of HR. Of course, many patients may have multifactorial HR due to a combination of mild ATIII deficiency and low heparin concentration. HR can usually be overpowered by administering higher doses of heparin, but there is no specific “maximal” dose which may be appropriately used. Heparin doses may need to be aggressively escalated to achieve a therapeutic effect [16].

Physiochemical characteristics are different among LMWHs because of the diverse methods of their manufacturing. The variations in molecular composition and pharmacological properties of LMWHs are reflected in differences in their clinical efficacy and safety. Tinzaparin is the only LMWH that is prepared by enzymatic hydrolysis with heparinase [17,18,19,20]. Due to its preparation method, tinzaparin owns distinct properties, including higher anti-IIa activity, lower anti-Xa/Anti-IIa activity ratio, higher release of tissue factor pathway inhibitor (TFPI), less dependence from renal function for its clearance, and more complete neutralization from its antidote, if needed. Increased thrombin generation (factor IIa) and tissue factor (TF) pathway activation are key pathological features in COVID-19-associated thrombosis [21]. In this context, special properties of tinzaparin, such as higher anti-IIa activity and higher TFPI production and release from endothelial cells, could have an expanded role beyond its well-known anticoagulant function. TFPI has also significant effects in various vascular, inflammatory, cardiovascular, hematological, and other disorders [22,23,24,25,26].

The aim of this study was to evaluate the overall clinical effectiveness and safety of higher than conventionally used prophylactic doses of anticoagulation with tinzaparin administered for VTE prevention in COVID-19 patients with moderate disease severity during hospitalization.

## 2. Materials and Methods

INTERACT (ClinicalTrials.gov: NCT05036824) is a retrospective, phase IV, observational, non-interventional cohort study that aimed to collect data regarding thromboprophylaxis management in high thrombotic risk hospitalized, non-ICU patients with COVID-19 pneumonia.

Data were retrospectively collected from high thrombotic risk patients who received thromboprophylaxis with tinzaparin, according to current clinical practice, during hospitalization, from April 2020 to November 2021 to evaluate the safety and efficacy of thromboprophylaxis and to examine possible associations of patients’ profiles with thrombotic and bleeding events and the course of illness. The study was conducted in Greece and a total of 13 hospitals participated in the study. There was no direct patient interaction in the study. The trial was approved by the relevant Institutional Review Boards and conducted in accordance with the Good Clinical Practice guidelines of the International Council for Harmonization and the Helsinki declaration.

High thrombotic risk patients were defined as patients hospitalized with COVID-19 and fever > 38 °C for >48 h plus one of the following: Age > 65 years, BMI ≥ 30 Kgr/m^2^, diabetes mellitus, hypertension, cardiovascular disease, respiratory disease, thrombosis history, known thrombophilia, history of immobility/limited mobility, active cancer (for which received treatment the last 6 months), recent surgery or trauma (last 3 months), and D-dimers level > ULN.

Moderate disease severity was defined as patients who were hospitalized with COVID-19 and who were not critically ill (which was defined as an absence of critical care–level of organ support at enrollment), e.g., patients with score 4 (hospitalized, no oxygen therapy) or 5 (hospitalized; oxygen by mask or nasal prongs) as assessed using the World Health Organization (WHO) progression scale.

Patients with a positive polymerase chain reaction test for SARS-CoV-2 (PCR+ SARS-CoV-2), from any specimen, admitted to hospital with COVID-19 infection and administered thromboprophylaxis with tinzaparin in an intermediate to full treatment dose, aged ≥18 years, who had signed consent, upon their admission, to allow the anonymized medical data of their hospitalization to be used for future research, enrolled in the study. Although there was not a specific protocol shared by the participating centers in terms of administered dose and centers were following their individual clinical practices, these practices were quite similar.

Patients who did not meet all criteria to be eligible, or were pregnant, had a current diagnosis or suspicion of pulmonary thromboembolism or deep vein thrombosis, or whose progression to death was imminent and inevitable within 24 h of their admission, irrespective of the provision of treatments, were excluded from our study. Patients with indication of chronic therapeutic anticoagulation were not included in the study. Note that participating centers did not screen patients for DVT at admission.

The primary objective of this study was to evaluate the current management approach with “intermediate” to “full therapeutic” doses of tinzaparin for thromboprophylaxis in non-critically ill hospitalized patients, with confirmed COVID-19, in terms of efficacy (incidence of objectively confirmed symptomatic distal deep vein thrombosis (DVT), symptomatic or incidental proximal DVT, symptomatic or incidental pulmonary embolism (PE), or both DVT and PE or fatal PE) and safety (incidence of any bleeding event, including major, clinically relevant non-major bleeding (CRNMB), and minor bleeding events). The secondary objective was to examine the course of illness of COVID-19 via measure of laboratory parameters (CRP (mg/dL), D-dimer (µg/L), Ferritin (ng/mL), hemoglobin (gm/dL), PLTs (Count/mcL)), SpO2 (%), as well as clinical improvement and/or survival, assessed at prespecified time points post admission. SpO_2_ was measured in patients breathing ambient or “room” air (FIO_2_ = 0.21). Clinical improvement and/or survival during hospitalization was assessed using the World Health Organization (WHO) progression scale [27].

An intermediate dose of tinzaparin was defined as 50–75% of the full therapeutic dose, subcutaneous (SC), once daily (OD). A therapeutic dose is 175 Anti-Xa IU/Kg of body weight, SC, OD. Both primary and secondary objectives were evaluated during hospitalization. Patients were otherwise treated with standard antiviral and anti-inflammatory regimens according to current national recommendations conforming to international guidelines at the time, i.e., remdesivir, dexamethasone +/− tocilizumab/baricitinib.

In all cases, laboratory and clinical parameters were collected at 3 time points: at admission, after one week ± two days and at discharge from the hospital. VTE events were objectively confirmed with internationally recommended imaging techniques [28,29]. For all patients, bleeding events were classified using those criteria recommended from the ISTH [30,31,32]. Additionally, we assessed the association of the panel of laboratory and clinical parameters representing an inflammatory and hemostatic state with VTE and bleeding events and clinical outcomes.

### Statistical Analysis

All collected data from the patients’ files were accumulated in a Microsoft Excel file (Microsoft Inc., Redmond, WA, USA) for subsequent conditioning and preprocessing. The SAS statistics software version 9.4 (SAS Institute Inc., Cary, NC, USA) was used to perform the statistical analysis. Descriptive statistics for data expressed in numeric format (arithmetic variables) were presented as the median value and the quartile 1 to quartile 3 (Q1–Q3) range. For the categorical data, the frequencies and the relevant percentages were used. Comparisons between groups, for the arithmetic variables were performed using the Mann–Whitney (MW) U test since the arithmetic variables were not produced from normal distribution (as evaluated by the Kolmogorov–Smirnov test). Categorical variables were examined using the chi-square test or Fisher’s exact test; the latter was used if the number of expected cases within the groups under comparison was <5 for more than 25% of the contingency table cells. For 2 × 2 contingency tables the odds ratios (ORs) along with the 95% confidence intervals (CI) were presented. *p* < 0.05 was considered as statistically significant for all cases, and all tests were two-sided.

## 3. Results

### 3.1. Study Population

A total of 705 patients from 13 COVID-19 clinics throughout Greece were included in the study; their baseline characteristics and medical history are presented in Table 1. The median age was 62 years (min: 18, max: 96, Q1–Q3: 49). Fifty-five percent of the patients were males; the median weight was 80 Kgr; the median height was 1.7 m (range 1.3–1.9 m, Q1–Q3: 1.65–1.78 m), and the median BMI: 27.3 Kgr/m^2^. There were no differences regarding age or BMI regarding men and women: 63 (50–74) vs. 60 (49–72) years, and 27.3 (25–30.9) vs. 27.2 (25.3–29.4) Kgr/m^2^, respectively. About 3% of the participants had a history of heart attack or stroke; eight patients had experienced more than one event. Two patients had a history of gastrointestinal tract bleeding. Common comorbidities included hypertension (261, 41.5%), diabetes mellitus (158, 25.4%), and ischemic heart disease (122, 18.1%). Smoking habits were reported for 107 (26.2%) patients.

### 3.2. Tinzaparin Dose and Duration

Anticoagulation with tinzaparin was initiated immediately upon patient hospitalization for most of the patients; only in 12 patients was tinzaparin administered later than the admission date. The once-daily dosage used was 8000 Anti-Xa IU in 31.8% of the patients, 10,000 Anti-Xa IU in 28.9% of the patients, and 14,000 Anti-Xa IU in 29.5% of them. The full therapeutic dose was received by 36.3% of the participants (mean ± SD: 166 ± 33 IU/Kgr/day), and the remaining patients (63.9%) received an intermediate dose (mean ± SD: 114 ± 22 IU/Kgr/day). In the majority of the patients (95.2%), the dose remained the same throughout the study; the dose was increased in 20 patients (2.8%) and decreased in 14 patients (2.0%). From the 20 patients in whom the dose was increased, 4 had presented thrombotic events, and for those remaining the dose was increased from 9625 ± 1500 IU/day to 13,750 ± 1770 IU/day due to an increase in the D-dimers level. For the 14 patients in whom the dose was decreased, the dose was adjusted from 14,428 ± 2243 to 10,571 ± 1650 IU/day due to quick and significant health improvement; none of these patients had experienced a bleeding event. The median duration of treatment was 13 days (Q1–Q3: 8–20 days) reflecting the hospitalization period.

### 3.3. Tinzaparin Effectiveness and Safety

In total, 14 thrombotic events (2.0%) were observed; of these, three (*n* = 3) were PE and eleven (*n* = 11) DVT. Eight of the thrombotic events were identified during the second checkpoint (i.e., one week ± two days after admission) while six events occurred after this evaluation point.

In terms of safety, four bleeding events were recorded (0.6%) in total. Two of these events were observed in patients who developed DVT; in one of these, anticoagulation continued with a reduced dose. There were no episodes of intra-cerebral bleeding or confirmed HIT.

In-hospital death occurred in 12 patients (1.7%) due to disease progression. One of these had simultaneous DVT and bleeding and another one presented with bleeding which was not attributed to anticoagulation. Table 2 summarizes the primary study outcomes.

### 3.4. Association of Laboratory and Clinical Parameters with Thrombosis

We examined potential associations of the demographic data, as well as the clinical and laboratory parameters, as these were expressed at the three checkpoints for the patients that experienced thrombotic events vs. the patients that did not experience thrombosis (the results are summarized in Table 3). Age was found to be an important risk factor, since patients with thrombosis were about 15 years higher in median age (*p* = 0.0149). Meanwhile, weight, height, and BMI were not found to have a role (*p* > 0.05).

From the possible predictors of thrombosis, during admission, the D-dimers level was higher in patients that developed thrombotic events as compared to those that did not develop such events (median (Q1–Q3): 2490 µg/L (1580–6480) vs. 700 µg/L (400–1475) receptively, *p* < 0.0001). One week ± two days after admission, the median D-dimers level in the group of patients that experienced thrombosis was found to be increased by 1020 µg/L, and there was still a statistically significant difference in comparison with the D-dimers level of patients without thrombotic events (D-dimers median: 619 µg/L, *p* = 0.000014). In contrast, the hemoglobin level of patients with thrombosis dropped and was significantly lower than the level of the group without thrombosis (median (Q1–Q3): 12.3 gm/dL (10.9–13.5) vs. 13.5 gm/dL (12.1–14.2), *p* = 0.0327). Finally at discharge, the D-dimers levels had decreased in comparison with the evaluation at one week ± two days but they were still higher in the patients that had thrombosis (median (Q1–Q3): 1618.5 µg/L (1010–2255) vs. 500 µg/L (294–918), *p* < 0.0001). In addition, ferritin was higher (median (Q1–Q3): 634 ng/mL (454.5–845) vs. 410 ng/mL (210–645), *p* = 0.0397) and the platelet count was lower (*p* = 0.0318); see Table 3 for details).

### 3.5. Evolution of Laboratory and Clinical Parameters

**Figure 1:**
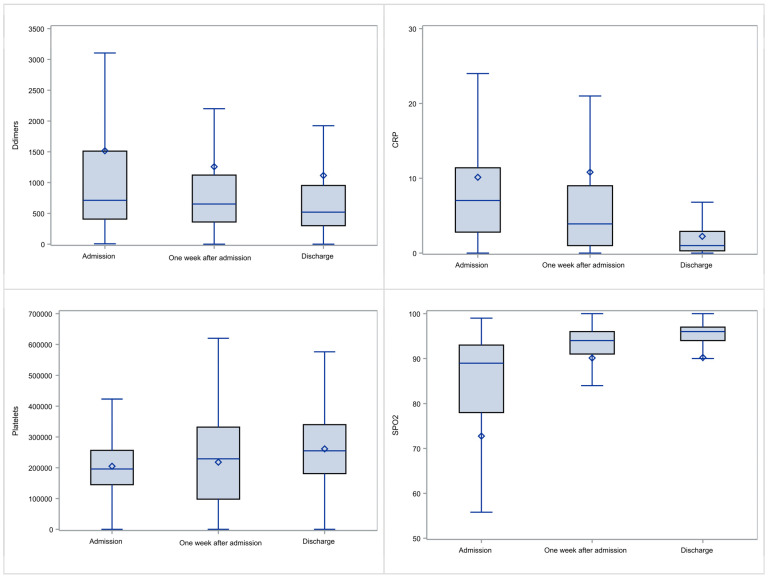
Evolution of D-Dimers, CRP, PLTs and SpO2 from admission to discharge. In each plot the lower and upper part of the boxes represent the Q1 and Q3 values; horizontal lines within the boxes correspond to the median values; the whisker limits correspond to the lower an upper value after excluding outliers; and the diamond symbols represent the mean value. Outliers are not presented.

For the total cohort, in relation to the evolution of the patients’ laboratory results (D-dimers, CRP and PLTs) and the SpO2 measurements, there were observed significant improvements over time (see Figure 1). Specifically, the D-Dimers median level for the total population was reduced from admission towards the first week ± two days from 712 µg/L to 652 µg/L, and subsequently to 520 µg/L upon discharge (*p* < 0.001 for the three time-points comparison, *p* < 0.0072 between admission and the first week ± two days and *p* = 0.0007 from the first week ± two days towards discharge). When a linear regression model was fitted to the data, on average D-dimers were reduced by 5 ± 14.4 µg/L per day (however *p* = 0.7235 and with poor adjusted R^2^).

The median CRP value for all patients was reduced from 7.0 mg/dL on admission to 3.9 mg/dL the first week ± two days and subsequently patients had median CRP 1.0 mg/dL at discharge from hospital (*p* < 0.0001 when comparing the three time points and *p* < 0.0001 between admission and one week ± two days as well as *p* < 0.0001 between one week ± two days and exit). A statistically significant linear regression model was not possible to be achieved (adjusted R^2^ = 0.6%).

Platelet levels were increased, specifically from 196,000/mcL (median) on admission to 229,000/mcL within one week ± two days and subsequently patients had median platelets count 255,000/mcL on discharge from hospital (*p* = 0.0005 from admission to first week ± two days and *p* = 0.0001 from first week ± two days to exit). Similarly, no appropriate fit for a linear regression model was obtained (R^2^ = 1.6%).

Finally, SpO2 was significantly improved from 89.0% (Q1–Q3: 78–93%) on admission (i.e., well below normal range) to 94.0% (Q1–Q3: 91–96%) on the first week ± two days and subsequently to normal levels 96% (Q1:Q3: 94–97%) on exit (*p* < 0.0001 in both cases).

The evolution of D-Dimers, CRP, PLTs and SpO2 values of the total cohort from admission to discharge is depicted in Figure 1.

**Figure 2:**
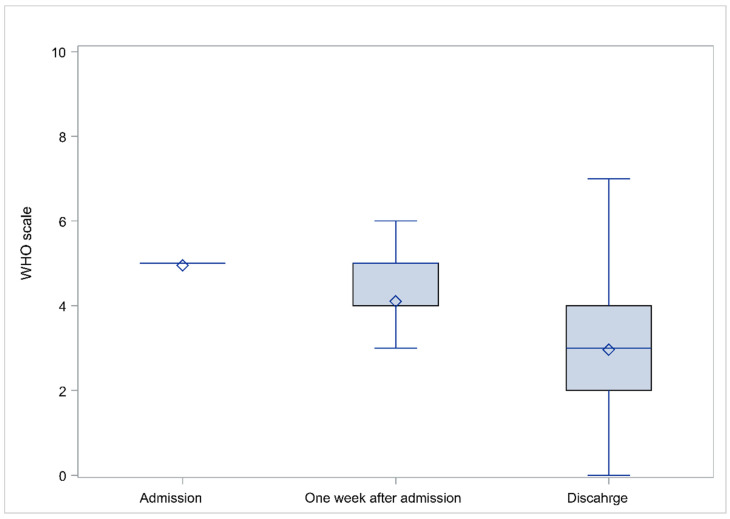
Evolution of patients’ health status from admission to discharge, as it is reflected by the WHO scale. In each plot the lower and upper part of the boxes represent the Q1 and Q3 values; horizontal lines within the boxes represent the median values; the whisker limits correspond to the lower and upper value after excluding outliers; and the diamond symbols represent the mean value. Outliers are not presented.

The evolution of patients’ course of illness and improvement as concluded by the WHO progression scale evaluation is presented in Figure 2. Clearly, the WHO progression scale value drops over time indicative of health improvement; however, the high values, as indicated by the whiskers, show that for some patients there were health deterioration.

## 4. Discussion

Our study included 705 non-critically ill patients hospitalized with COVID-19, receiving an intermediate to full therapeutic-dose of anticoagulant prophylaxis with tinzaparin. For most patients (95.2%) the dose remained unchanged throughout the study period. The median duration of treatment was 13 days, reflecting the hospitalization period. In total, 14 thrombotic (2.0%) and four bleeding events were observed (0.6%) during the observation period. Age was found to be an important risk factor for developing thrombosis and D-dimer levels at admission were higher in patients that developed thrombotic events. In-hospital death occurred in 12 patients (1.7%) due to disease progression. For the total cohort, in relation to the evolution of the patients’ laboratory results (D-dimers, CRP, and PLTs), and the SpO2 measurements observed significant improvements over time. For the majority of patients, the WHO progression scale score dropped over time indicating health improvement.

Data suggest that thromboembolic event rates are higher in COVID-19 compared to other viral infections and appear to increase in proportion to the severity of illness [33]. Similar to other viral infections, VTE is the most common vaso-occlusive event in COVID-19, with DVT and PE predominating [34]. Incidence of VTE events has been found to be up to 8.4% in non-critically ill and up to 18.6% in critically ill patients [35].

Despite ongoing investigation of anticoagulant prophylaxis optimization in COVID-19 patients, as well as emergence of clinical guidelines, not all areas are clearly addressed [36]. Although all guidelines recommend starting anticoagulation for venous thromboprophylaxis in all hospitalized patients with COVID-19, preferably with LMWH, they currently represent living guidance in view of the results of randomized clinical trials. Open questions remain regarding the choice of agent and optimal dosing of anticoagulation based on or irrespective of illness severity, as well as the utility of VTE prophylaxis after hospital discharge.

Recently, a big multiplatform clinical trial was performed with the combined populations of three separate investigations: REMAP-CAP, ACTIV 4a, and ATTACC [37]. In this trial, patients who were hospitalized with COVID-19 and who were not critically ill were randomized to receive pragmatically defined regimens of either therapeutic-dose anticoagulation with heparin or usual-care pharmacologic thromboprophylaxis. The primary outcome was organ-support-free days. Secondary efficacy outcomes included, among others, survival until hospital discharge, a major thrombotic event, or death and any thrombotic event including deep venous thrombosis. Secondary safety outcomes that were assessed during the treatment period were major bleeding and laboratory-confirmed heparin-induced thrombocytopenia (HIT) [37]. The demographic and clinical characteristics of the patients at baseline in the group receiving therapeutic doses in this multiplatform trial were quite similar to those in our cohort. In both cases, a significant proportion of patients had comorbidities, with the most frequent reported to be hypertension (53.4% vs. 41.5%), diabetes mellitus (29.8% vs. 25.4%), and heart disease (10.6% vs. 18.1%) in the clinical trial and our cohort, respectively. Notably, age > 65 years, male gender, arterial hypertension, diabetes mellitus, cardiovascular disease (CVD), and respiratory disease are significant risk factors for severe COVID-19, disease worsening, and death [38]. The conclusion of the multiplatform clinical trial was that therapeutic anticoagulation increased survival in non-critically ill hospitalized COVID-19 patients. Survival until hospital discharge was 92.7% in a multiplatform trial, while in our analysis it was 98.3%, since in-hospital death occurred in 12 out of 705 patients included. Notably, in the multiplatform trial, in the therapeutic anticoagulation group only 79.6% of the patients received a full therapeutic dose while in the usual-care pharmacological thromboprophylaxis group 28.3% of the patients received higher than low dose thromboprophylaxis.

In the ACTION trial, in patients hospitalized with COVID-19 with elevated D-dimer concentration, initial in-hospital therapeutic anticoagulation with rivaroxaban for stable patients or enoxaparin for unstable patients, followed by rivaroxaban through 30 days, did not improve clinical outcomes, and increased bleeding compared with in-hospital prophylactic anticoagulation. Authors concluded that the use of therapeutic-dose rivaroxaban, and other direct oral anticoagulants, should be avoided in hospitalized patients with COVID-19 who do not have an evidence-based indication for oral anticoagulation [39]. In the RAPID trial, in moderately ill patients with COVID-19 and increased D-dimer levels admitted to hospital wards, therapeutic dosages were not associated with an increased bleeding risk but did not improve the composite primary outcome (death, invasive mechanical ventilation, non-invasive mechanical ventilation, or ICU admission) compared to prophylactic dosages in moderately ill ward patients with D-dimers two times the upper limit of normal. Indeed, there was only a reduction in death from any cause, with no difference in bleeding risk. However, there was no difference in VTE, mechanical ventilation, or ICU admission either [40]. Outcomes seemed to differ with disease severity (critically ill versus moderately ill); initial use or escalation to intermediate dosing did not improve a composite outcome of thrombosis or mortality in COVID-19 ICU patients [41,42].

Retrospective studies have also produced mixed results underlying the need for further randomized clinical trials [43,44,45]. In a retrospective study by Jonmarker et al., evidence supported the use of a high dose of tinzaparin or dalteparin for thromboprophylaxis for critically ill patients, showing reduction in mortality without major bleeding events [46].

Confirmed VTE events occurring during index hospitalization were 16 (10 PE and 6 DVT)/1180, 1.36% in REMAP-CAP, ACTIV 4a and ATTACC multiplatform trial vs. 14 (3 PE and 11 DVT)/705, 2% in our cohort [37]. Notably, thrombotic events in our analysis were found to be associated with age and higher D-dimer levels at admission. However, since participating centers did not screen patients for DVT at admission, the possibility of asymptomatic VTE presence at that time point cannot be excluded.

A major bleeding event occurred in 22/1180 (1.9%) in the REMAP-CAP, ACTIV 4a, and ATTACC trial while four bleeding events were observed (0.6%) in our total population; one of these was major. There were no episodes of intra-cerebral bleeding or confirmed HIT [37].

Potential mechanisms for the increase in D-dimer levels in patients with COVID-19 include pulmonary endothelial injury with inflammation-associated deposits of intra-alveolar fibrin, systemic endothelial injury with diffuse thrombosis of smaller vessels [47] or of larger veins [48], and coagulopathy. A systematic review reported the mean D-dimer level to be 580 μg/L in 1551 patients with mild disease and 3550 μg/L in 708 patients with severe disease [49]. Additionally, a meta-analysis showed that patients with elevated D-dimer on admission had a higher risk of mortality (relative risk, RR 1.82) and disease severity (RR 1.58) compared to patients with normal levels of D-dimer [50]. Obviously, the in-hospital course of D-dimer may reflect disease activity in COVID-19 patients. D-Dimers median level for the total population in our cohort, was reduced from admission towards the first week ± two days from 712 µg/L to 652 µg/L and subsequently to 520 µg/L upon discharge (*p* < 0.001 for the three-time points comparison) indicating a decrease in disease activity. Moreover, incidence of in-hospital death due to disease progression was low (1.7%).

A recent systematic review of 60 observational plus one case-control studies, comprised of 13,891 COVID-19 patients from fifteen countries, demonstrated that the severe cases had higher levels of C–reactive protein when compared to the mild cases in all studies (100%). The increase in C-reactive protein was statistically significant in 78.7% of the cases. Authors concluded that high levels of CRP are associated with COVID-19 severity [51]. The median CRP value for all patients in our analysis was reduced from 7.0 mg/dL on admission to 3.9 mg/dL the first week ± two days, and subsequently to 1.0 mg/dL during discharge from hospital (*p* < 0.0001 when comparing the three time points) indicating an improvement in terms of disease severity. However, a statistically significant linear regression model was not possible to achieve.

Early in the pandemic it became evident that SARS-CoV-2 can directly or indirectly attack some blood cells such as platelets (PLT). About a quarter of COVID-19 patients have experienced thrombocytopenia (PLT less than 150,000/mcL), especially in the first week after admission to the hospital [52]. It should be mentioned that thrombocytopenia is not always an early event in COVID-19, as a considerable number of patients may experience it during disease progression several days after infection [53]. Either induced early or delayed, thrombocytopenia can prolong patients’ hospitalization, increase their need for ventilation, and increase the 28-day mortality risk for patients [54]. The lower the PLT level is, the poorer is the outcome and the higher the risk of mortality [55]. The evolution of platelet levels in our total cohort from 196,000/mcL (median) on admission to 229,000/mcL within one week ± two days and subsequently to 255,000/mcL on discharge from hospital (*p* = 0.0001 from first week ± two days to exit) again indicated a decline in disease severity; similarly, with CRP, no appropriate fit for a linear regression model was obtained.

In terms of ferritin and hemoglobin levels, no significant variations were observed during the course of hospitalization.

The above-mentioned improvements were also reflected in clinical parameters; specifically, SpO_2_ was significantly improved from 89.0% on admission to 94.0% in the first week ± two days and, subsequently, to normal levels; 96% on exit from hospital (*p* < 0.0001). At the same time, patients’ course of illness and severity, as this is concluded by the WHO progression scale evaluation, dropped over time, which is indicative of health improvement. However, the scale has challenges, especially at the lower end of the scale where the measures are more subjective. At the upper end of the scale, the use of support measures is variable, reflecting not only on the patient’s baseline comorbidities but also on the local practice preferences.

Because SARS-CoV-2 infection incites an inflammatory response that may lead to hypercoagulability [56] and potentially contribute to organ failure [57,58,59], heparins could probably improve the course of illness, not only through antithrombotic but also through anti-inflammatory and potentially antiviral mechanisms [15].

The LMWHs vary in their physicochemical properties, the anti-Xa/anti-IIa ratio, and their inhibitory effect on thrombin generation [60,61]. Tinzaparin is the only LMWH produced by enzymatic depolymerization [62]. Due to its method of production, it has a higher mean molecular weight in comparison with other LMWHs [63]. The longer chain length of tinzaparin translates into greater inhibition of coagulation factor IIa (thrombin), greater release of tissue factor pathway inhibitor (TFPI), and elimination by both renal clearance and the reticuloendothelial system, making it less likely that tinzaparin accumulates in elderly and patients with renal impairment [61,64,65,66]. Apart from its anticoagulant role, TFPI also has a role in other important processes such as inflammation [67].

Considering the key role of increased thrombin generation (factor IIa) and tissue factor (TF) pathway activation in COVID-19-associated thrombosis [21], those features of tinzaparin abet the hypothesis for tinzaparin to have an extended role, interfering not only with coagulation cascade but also exhibiting anti-inflammatory potency in treatment and prophylaxis for COVID-19 patients [68].

Our study has the limitations and advantages of an observational study. For example, the study was designed in a broad range of routine clinical practices in COVID-19 clinics, without specific focus on patients’ characteristics; thus, unknown bias could be introduced. There was no selection of patients into intervention and control groups. Additionally, all the data were gathered from medical records by chart review of the individual patients in a retrospective fashion. Since disease progression, clinical outcome, and biochemical outcome is very complex and additional factors, such as other drugs and general clinical care, are very important to drive improved outcome, adequate thromboprophylaxis is just a part of the management of patients with COVID-19. However, in the author’s opinion, this study captured the real-life conditions in a routine clinical setting in a COVID-19 department. The present cohort includes practice-based evidence in hospitalized patients with moderate disease severity. Additional strengths of our approach were the number of patients included in the analysis as well as the evaluation of single anticoagulant modality.

## 5. Conclusions

Among non-critically ill patients hospitalized with COVID-19, prophylactic anticoagulation with intermediate (63.7%) to therapeutic (36.3%) dosages of tinzaparin were safe and seemed effective. Together with standard-of-care antiviral treatment, there were improvements in both laboratory and clinical parameters reflecting a decrease in disease severity during the course of illness, as well as a drop in the WHO progression scale over hospitalization time, indicating health improvement. Survival until hospital discharge was 98.3%. Further studies need to be performed and more data supporting intensive doses of anticoagulation are required.

## Figures and Tables

**Table 1:** Demographic data, past medical history, and anticoagulant treatment prior to admission of the study population.

Characteristic	Measure
Age (years) median (Q1–Q3 range)	62 (49–72.5)
Gender (male) N (%)	390 (55.2%)
Weight (Kgr) median (Q1–Q3 range)	80 (72–89)
Height (m) median (Q1–Q3 range)	1.7 (1.65–1.78)
BMI (Kgr/m^2^) median (Q1–Q3 range)	27.3 (25.2–30.1)
Smoking N (%)	107 (26.2%)
Thrombosis history N (%)	30 (5%)
Heart attack	11
Stroke	10
Deep Venous Thrombosis (DVT)	4
Arterial Thrombotic Events (ATE)	2
Superficial Vein Thrombosis (SVT)	2
Type not reported	1
Bleeding history N (%)	2 (0.4%)
Immobility history N (%)	37 (8%)
Varicose veins N (%)	17 (3.7%)
Family history of thrombosis N (%)	2 (0.4%)
History of central catheter placement N (%)	5 (1.1%)
Inherited thrombophilia N (%)	1 (0.3%)
Recent surgery N (%)	5 (1.2%)
Heart disease N (%)	122 (18.1%)
Hypertension N (%)	261 (41.5%)
Diabetes N (%)	158 (25.4%)
Renal insufficiency N (%)	24 (3.4%)
Liver disease	None
Inflammatory bowel disease N (%)	18 (2.9%)
Endocrine disorders N (%)	80 (12.9%)
Respirator problems N (%)	58 (8.7%)
Malignancies N (%)	11 (2.2%)
Other comorbidities N (%)	100 (18.3%)
Long-term use of DOAC or acenocoumarol N (%)	22 (5.0%)
Long-term use of heparins (history) N (%)	3 (0.1%)
Long-term use of antiplatelet or aspirin N (%)	56 (14.9%)

**Table 2:** Primary outcomes of INTERACT study.

	Primary Outcome	N	%
Efficacy	Symptomatic distal deep vein thrombosis (DVT)	11	1.6%
	Symptomatic or incidental pulmonary embolism (PE)	3	0.4%
	Both DVT and PE	0	-
	Fatal PE	0	-
	Total	14	2.0%
Safety	Major	1	0.1%
	Clinically relevant non-major bleeding (CRNMB)	0	-
	Minor	3	0.4%
	Total	4	0.6%
	In hospital deaths	12	1.7%

**Table 3:** Demographic, clinical, and laboratory data of the study population according to the development of thrombosis at the three checkpoints.

		Patients with Thrombosis (*n* = 14)	Patients without Thrombosis (*n* = 691)	
	Characteristic	Median (Q1–Q3)	Median (Q1–Q3)	*p*
Demographics	Age (years)	74.5 (62–79)	61.9 (49–72)	0.0149
	Weight (Kgr)	74.5 (70–97)	80 (72–89)	0.9255
	Height (meter)	1.7 (1.6–1.8)	1.7 (1.7–1.8)	0.5368
	BMI (Kg/m^2^)	29.2 (24.2–33.2)	27.3 (25.2–30.1)	0.3417
Admission	CRP (mg/dL)	7.3 (3.7–13.6)	7.0 (2.8–11.4)	0.6487
	D-dimers (µg/L)	2490 (1580–6480)	700 (400–1475)	<0.0001
	Ferritin (ng/mL)	429 (297–722)	508 (278–870)	0.7281
	Hemoglobin (gm/dL)	12.9 (11.4–14.5)	13.6 (12.2–14.6)	0.2871
	PLTs (Count/mcL)	221,500 (164,000–340,000)	195,500 (145,000–255,000)	0.1357
	SpO2 (%)	91 (87–96)	89 (78–93)	0.2258
	Tinzaparin dose (Anti-Xa IU)	11,000 (10.000–14,000)	10,000 (8000–14,000)	0.0398
	WHO progression scale	5 (5–5)	5 (5–5)	0.5311
One week ± two days after admission	CRP (mg/dL)	6.7 (4–12.5)	3.9 (1–9)	0.0704
	D-dimers (µg/L)	3510 (1458–9500)	619 (352–1054.5)	<0.0001
	Ferritin (ng/mL)	770 (274–1047)	542 (299–942)	0.4253
	Hemoglobin (gm/dL)	12.3 (10.9–13.5)	13.5 (12.1–14.2)	0.0327
	PLTs (Count/mcL)	266,500 (130,500–366,000)	227,000 (98,000–332,000)	0.4367
	SpO2 (%)	92 (88.5–97)	94 (91–96)	0.6408
	Tinzaparin dose (Anti-Xa IU)	14,000 (10,000–18,000)	10,000 (10,000–14,000)	0.0608
	WHO progression scale	5 (5–5)	5 (4–5)	0.0769
Discharge	CRP (mg/dL)	0.6 (0.2–2.6)	1 (0.3–2.9)	0.6361
	D-dimers (µg/L)	1618.5 (1010–2255)	500 (294–918)	<0.0001
	Ferritin (ng/mL)	634 (454.5–845)	410 (210–645)	0.0397
	Hemoglobin (gm/dL)	12.6 (10.7–14.1)	13.3 (12–14.2)	0.2379
	PLTs (Count/mcL)	176,000 (127,000–280,000)	255,000 (183,000–340,000)	0.0318
	SpO2 (%)	96 (92–97)	96 (94–97)	0.9332
	Tinzaparin dose (Anti-Xa IU)	14,000 (14,000–18,000)	10,000 (10,000–14,000)	0.0224
	WHO progression scale	4 (4–5)	3 (2–4)	0.0073

## Data Availability

INTERACT Study data are available from the corresponding author upon reasonable request.

## Appendix A. INTERACT Study Group

Karolina Akinosoglou, Internal Medicine Department, University General Hospital of Patras, akin@upatras.gr

Christos Savopoulos, 1st Propaedeutic Department of Internal Medicine, University General Hospital of Thessaloniki “AXEPA”, chrisavopoulos@gmail.com

Abraham Pouliakis, 2nd Department of Pathology, National and Kapodistrian University of Athens, “ATTIKON” University Hospital, apou1967@gmail.com

Charalampos Triantafyllidis, 1st Pulmonology Department, General Hospital of Kavala “Saint Sylas”, harrydoc2000@yahoo.gr

Eleftherios Markatis, Pulmonary Department, General Hospital of Corfu “Saint Eirini”, lefte_mark83@yahoo.gr

Foteini Golemi, Pathology Department, Argolida General Hospital-Argos Hospital Unit, Foteinh_golemi@hotmail.com

Angelos Liontos, Department of Internal Medicine, Faculty of Health Sciences, School of Medicine, University of Ioannina, Ioannina, Greece angelosliontos@gmail.com

Charikleia Vadala, 4th Internal Medicine Department, Athens General Hospital “Evangelismos”, hara.vadala@gmail.com

Ilias Papanikolaou, Pulmonary Department, General Hospital of Corfu “Saint Eirini”, icpapanikolaou@hotmail.com

Vasiliki Dimakopoulou, Internal Medicine Department, University General Hospital of Patras, dimakopoulou.vasilina@gmail.com

Panagiotis Xarras, Internal Medicine Department, General Hospital of Kozani “Mamatseio”, panosxarras@gmail.com

Katerina Varela, Pulmonary Department, General Hospital of Patras “Saint Andreas”, alerav99@yahoo.gr

Georgia Kaiafa, 1st Propaedeutic Department of Internal Medicine, University General Hospital of Thessaloniki “AXEPA”, gdkaiafa@yahoo.gr

Athanasios Mitsianis, Internal Medicine Department, General Hospital of Ptolemaida “Bodosakeio”, athemit@gmail.com

Anastasia Chatzistamati, Internal Medicine Department, General Hospital of Kastoria, 5755anastasiachtz@gmail.com

Efthalia Randou, Internal Medicine Department, General Hospital of Kozani “Mamatseio”, vag_roundos@yahoo.gr

Spyridon Savvanis, Internal Medicine Department, General Hospital of Athens “Elpis”, savvanis@live.com

Maria Pavlaki, Pathology Department, Argolida General Hospital-Argos Hospital Unit, mar_paul300@yahoo.gr

Georgios Efraimidis, Internal Medicine Department, General Hospital of Patras “Saint Andreas”, efrgeorge@gmail.com

Vasileios Samaras, Internal Medicine Department, General Hospital of Kastoria, billsamaras@gmail.com

Dimitrios Papazoglou, Infectious Diseases Unit, 2nd Department of Internal Medicine, University General Hospital of Alexandroupolis, dpapazog@med.duth.gr

Alexandra Konstantinidou, 1st Pulmonology Department, General Hospital of Kavala “Saint Sylas”, konstantinidoua4@gmail.com

Periklis Panagopoulos, Infectious Diseases Unit, 2nd Department of Internal Medicine, University General Hospital of Alexandroupolis, ppanago@med.duth.gr

Haralampos Milionis, Department of Internal Medicine, Faculty of Health Sciences, School of Medicine, University of Ioannina, Ioannina, Greece, hmilioni@uoi.gr

Michail Anthopoulos, 1st Propaedeutic Department of Internal Medicine, University General Hospital of Thessaloniki “AXEPA”, mixalisonco@gmail.com

Emanouil Antonakis, Pulmonary Department, General Hospital of Corfu “Saint Eirini”, manos24m@yahoo.gr

Vasiliki Theodora Argyriadou, 1st Pulmonology Department, General Hospital of Kavala “Saint Sylas”, D.Argyriadou@yahoo.gr

Fotios Barkas, Department of Internal Medicine, Faculty of Health Sciences, School of Medicine, University of Ioannina, Ioannina, Greece; fotisbarkas@windowslive.com

Dimitrios Biros, Department of Internal Medicine, Faculty of Health Sciences, School of Medicine, University of Ioannina, Ioannina, Greece; md06238@uoi.gr

Eleni Karlafti, 1st Propaedeutic Department of Internal Medicine, University General Hospital of Thessaloniki “AXEPA”, linakarlafti@hotmail.com

Artemis Kyriakidou, Internal Medicine Department, General Hospital of Kastoria, artemiskir@gmail.com

George Marakomichelakis, 4th Internal Medicine Department, Athens General Hospital “Evangelismos”, gmarakom@gmail.com

Markos Marangos, Internal Medicine Department, University General Hospital of Patras, maragos@upatras.gr

Georgia Mpasioti, Internal Medicine Department, General Hospital of Patras “Saint Andreas”, geobas@hotmail.gr

Christos Mpozinis, Internal Medicine Department, General Hospital of Kastoria, christosboz5@gmail.com

Memet Noursen, 1st Pulmonology Department, General Hospital of Kavala “Saint Sylas”, dr_noursen@hotmail.com

Elisavet Papadopoulou-Skordou, Internal Medicine Department, General Hospital of Kastoria, elpap96@gmail.com

Alkmini Penou, 1st Pulmonology Department, General Hospital of Kavala “Saint Sylas”, alkpeno@yahoo.gr

Vassilios Petrakis, Infectious Diseases Unit, 2nd Department of Internal Medicine, University General Hospital of Alexandroupolis, vasilispetrakis1994@gmail.com

Stefanos Potonos, 1st Pulmonology Department, General Hospital of Kavala “Saint Sylas”, potonos@gmail.com

George Schinas, Medical School, University of Patras, georg.schinas@gmail.com

Anastasios Semertzidis, Internal Medicine Department, General Hospital of Kastoria, tasossem@gmail.com
